# Prevalence and associated factors of pediatric emergency mortality at Tikur Anbessa specialized tertiary hospital: a 5 year retrospective case review study

**DOI:** 10.1186/s12887-018-1287-4

**Published:** 2018-10-02

**Authors:** Gemechu Jofiro, Kemal Jemal, Lemlem Beza, Tigist Bacha Heye

**Affiliations:** 1 Addis Ababa Regional Health Bureau Department of Emergency, Box 245, Addis Ababa, PO Ethiopia; 2 Department of Nursing, Salale University College of Health Sciences, Fitche, Ethiopia; 3 Department of Emergency Medicine, Addis Ababa University College of Health Sciences, School of Medicine, Addis Ababa, Ethiopia; 4 Department of Pediatric and Child Health, Division of Emergency Medicine and Critical Care, Addis Ababa University College of Health Sciences, School of Medicine, Addis Ababa, Ethiopia

**Keywords:** Incidence, Pediatrics mortality, Emergency department, Ethiopia

## Abstract

**Background:**

Childhood mortality remains high in resource-limited third world countries. Most childhood deaths in hospital often occur within the first 24 h of admission. Many of these deaths are from preventable causes. This study aims to describe the patterns of mortality in children presenting to the pediatric emergency department.

**Methods:**

This was a five-year chart review of deaths in pediatric patients aged 7 days to 13 years presenting to the Tikur Anbessa Specialized Tertiary Hospital (TASTH) from January 2012 to December 2016. Data were collected using a pretested, structured checklist, and analyzed using the SPSS Version 20. Multivariate analysis by logistic regression was carried out to estimate any measures of association between variables of interest and the primary outcome of death.

**Results:**

The proportion of pediatric emergency department (PED) deaths was 4.1% (499 patients) out of 12,240 PED presentations. This translates to a mortality rate of 8.2 deaths per 1000 patients per year. The three top causes of deaths were pneumonia, congestive heart failure (CHF) and sepsis. Thirty two percent of the deaths occurred within 24 h of presentation with 6.5% of the deaths being neonates and the most common co-morbid illness was malnutrition (41.1%).

Multivariate analysis revealed that shortness of breath [AOR=2.45, 95% CI (1.22-4.91)], late onset of signs and symptoms [AOR=3.22, 95% CI (1.34-7.73)], fever [AOR=3.17, 95% CI (1.28-7.86)], and diarrhea [AOR=3.36, 95% CI (1.69-6.67)] had significant association with early mortality.

**Conclusion:**

The incidence of pediatric emergency mortality was high in our study. A delay in presentation of more than 48 hours, diarrheal diseases and shortness of breath were significantly associated with early pediatric mortality. Early identification and intervention are required to reduce pediatric emergency mortality.

## Background

Child mortality rates remain high globally [1] with around 3.1 million neonates, 2.3 million infants and 2.3 million childhood deaths occurring every year [2]. Mortality rates in children younger than 5 years have dropped from 11.9 million deaths in 1990 to 7.7 million deaths in 2010 [2]. Worldwide, the distribution of deaths in children fewer than five years of age is 33% in south Asia, 50% in sub-Saharan Africa, and less than 1% in high-income countries [3]. Common factors associated with childhood mortality include acute trauma, extremely preterm birth, and late presentation to the emergency units [4]. In resource-poor countries, pneumonia and diarrhea account for 20% of deaths in children fewer than 5 years old [2]. Malaria, AIDS, acute respiratory-tract infection, measles, and malnutrition were significantly contributed to child mortality [5]. In developing countries 10 to 20% of severely sick children are admitted to hospital every year [6–8].

In Africa, the childhood mortality rate is 92 per 1000 live births which are 15 times more than that of well-resourced countries [9]. Most childhood deaths from preventable communicable diseases and malnutrition were related to poor environmental health, poverty and lack of knowledge [10].

The magnitude and severity of child mortality are exacerbated by different factors, including delays in seeking assessment and treatment, diarrhea, and poor nutritional status [11]. In pediatric departments, early child mortality is commonly caused by preventable and reversible diseases, so urgent treatment and resuscitation are required to avoid poor outcomes [12, 13]. Early identification and treatment of pneumonia, sepsis, malaria, heart failure (secondary to anemia), acute respiratory tract infections, and diarrheal diseases has been shown to reduce childhood mortality in acute pediatric hospitals [14–17]. Effective intervention and good emergency care of children requires effort and coordination starting from the bedside up to the governmental level. Critical clinical issues, such as shortness of breath, fast breathing and fever with seizure are some of the preventable causes contributing to childhood mortality [12]. Despite advances in public health systems in Ethiopia through global partnerships, there is still a lack of well-organized pediatric emergency units. There is also limited information regarding pediatric mortality patterns, causes and associated factors [18].

Mortality rate is a reflection of the severity of illness and the quality of treatment of patients in pediatric emergency departments. The risk factors associated with the mortality of pediatric age groups in developing countries are largely unknown. This study aims to provide baseline pediatric mortality and valuable associated data essential to health care providers and administrators. This will help them allocate resources to the development of interventions, effective prevention and community education programs to reduce preventable childhood deaths in Ethiopia.

## Method

### Study design and period

This is a five-year retrospective chart review of cases presenting to an urban emergency department (ED) between January 1, 2012 and December 30, 2016.

### Study area

Tikur Anbessa Specialized Tertiary Hospital (TASTH) is an eight hundred bed hospital in Addis Ababa, Ethiopia. It services the most critical referred patients throughout the country. The pediatric emergency department had 42 beds and sees approximately 13,300 presentations per year. It was staffed by two pediatric emergency medicine specialists, residents, and 46 nurses.

### Inclusion criteria

Study data include pediatric patients aged 7 days to 13 years who died in the pediatric ED during the study period.

### Exclusion criteria

Pediatric patients aged 7 days to 13 years who died in the intensive care unit (ICU), neonatal care unit (NICU), or pediatric ward were excluded from the dataset. Patients with incomplete documentation were also excluded.

### Data collection

Data were collected by trained professional nurses using a pre-tested data collection form, which was adopted from previous similar studies [19–22]. Data collected includes socio-demographic characteristics, mode of transportation, clinical presenting features, and the main medical cause of mortality.

Age was categorized into four groups: i) neonate (7 to 28 days), ii) infant (one month to one year), iii) pre-school (one year to five years), and iv) school age (five years to thirteen years) [19, 20].

Referral sources were categorized into: i) internal health institution, ii) external health institution, and iii) self-referral [22].

Clinical data included nutrition status, episode of diarrhea within last year, previous hospital visit and/or admission within the last year, and type and duration of clinical presenting signs and symptoms. Nutritional status of the study participants was grouped into well-nourished and malnourished (mild, moderate, and severe) [22].

The outcome (pediatric mortality) was classified based on early mortality (death within 24 h of arrival to the ED) and late death (death more than 24 h after arrival to the ED) [19, 20].

Finally, the causes of mortality were defined according to the health management information system (HMIS) and international disease classification (IDC) at the hospital level across the country with related different pediatric age divisions [21].

Raw data on the causes and associated factors of pediatric emergency mortality were obtained from a secondary data source (HMIS registration books, medical chart or patient folder sheet, clinical care notes, and the hospital death certificate).

### Data processing and analysis

Data were analyzed using Statistical Package for Social Science (SPSS) version 20. Description of means, simple frequencies, proportions, and rates of the given data on each variable was calculated. Binary logistic regression was assessed to determine the relationship and association between dependent and independent variables. Crude odds ratios from bivariate logistic regression and adjusted odds ratios from multivariate logistic regression were calculated for potential confounding factors between the variables. A *p*-value of less than 0.05 was considered statistically significant and adjusted odds ratios with 95% confidence interval (CI) were calculated to determine strength of association.

### Ethical consideration

Ethical clearance and approval were obtained from the Ethical Committee of the Department of Emergency Medicine, College of Health Science, School of Medicine, Addis-Ababa University. Official letter was obtained from the Department of Emergency Medicine to the clinical director of TASTH. The ethical approval was received from the ethical committee for verbal consent from pediatric emergency department and Card Room staff before joining the study. Confidentiality was maintained in each level of the response. In view of the retrospective nature of this study and the secondary use of data from the health management information system database, study participant and family member consent was waived

## Results

Over the five-year study period, a total of 12,240 children (7 days old to 13 years old) presented to the pediatric emergency unit; 499 (4.1%) deaths were recorded. Of these, 338 (67.7%) pediatric deaths fulfilled the criteria for analysis, while the remaining 161 (32.3%) records were excluded because of incomplete documentation.

Table 1 lists the frequency distribution of socio-demographic characteristics and clinical presenting features of the study participants. More deaths occurred in males (56.5%), with a male to female ratio of 1.3:1. The average age was 37.5(±standard deviatio*n* = 43.2) months. Nearly half of the participants came from Addis Ababa region with more than 92.6% referrals from different health institution. Half of the study patients had previously visited a hospital, and more than 90% patients had a history of a hospital admission with different medical causes. Of all the deaths analyzed for this study, only 17.8% patients had a history of previous diarrhea within last year, and around 26.9% had history of malnutrition.

**Table 1:** Distribution of socio-demographic characteristics and clinical presenting features of study participants in PED at TASTH, Addis Ababa from 2012 to 2016 inclusive

Variables (*n* = 338)	Frequency	Percentage (%)
Sex		
Male	191	56.5
Female	147	43.5
Age category		
Neonate	69	20.4
Infant	92	27.2
Pre-school age	98	29.0
School age	79	23.4
Respondent residence		
From Addis Ababa	164	48.5
Out of Addis Ababa	174	51.5
Source of referral		
From health institution (internal and external)	313	92.6
Self-referral	25	7.4
Previous hospital visits within last year		
Yes	165	48.8
No	173	51.2
Previous hospital admission within last year		
Yes	152	92.1
No	186	7.9
Previous episode of diarrhea within last year		
Yes	60	17.8
No	278	82.2
Previous Nutritional status		
Normal	247	73.1
Malnourished	91	26.9
Duration of signs& symptoms		
≤ 2 days	134	39.6.0
> 2 days	204	60.4

**Fig. 1:**
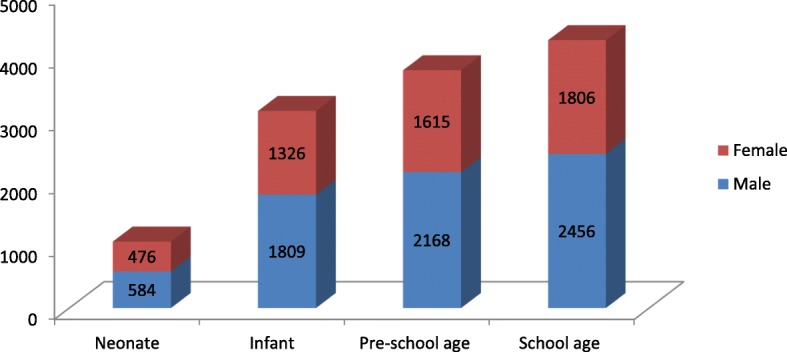
Sex identification among pediatric age group division in PED at TASTH, Addis Ababa from 2012 to 2016 inclusive

**Fig. 2:**
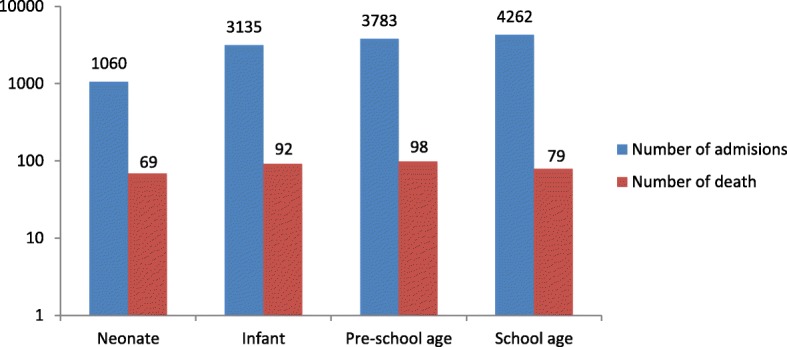
Age categories with mortality rate and number of admissions in PED at TASTH, Addis Ababa from 2012 to 2016 inclusive

Approximately 32% of deaths were documented as early death (within **≤**24 h of arrival in the pediatric emergency department). More than half (59%) patients presented for treatment following at least two days of signs and/or symptoms (Table 1). In all the age groups, males were admitted more often than females (Fig. 1), with higher numbers of deaths occurring in the pre-school age and infant age group (Fig. 2). However, the highest mortality rate was seen in the neonatal age group (6%), followed by infants (2.9%), then the other age groups (Fig. 2).

**Fig. 3:**
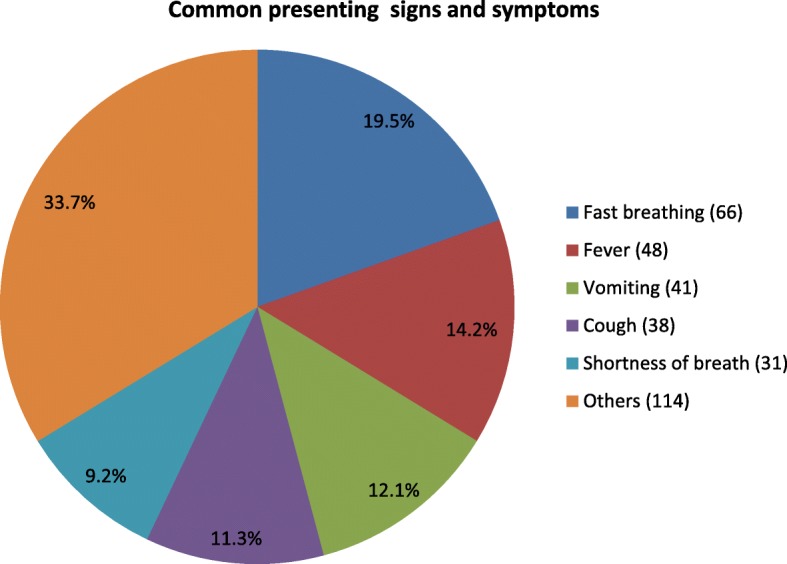
Clinical common presenting symptoms of study participants in PED at TASTH, Addis Ababa from 2012 to 2016 inclusive

The five most common presenting symptoms were fast breathing (66, 19.5%), fever (48, 14.2%), vomiting (41, 12.1%), cough (38, 11.2%), and shortness of breath (31, 9.2%) (Fig. 3).

### Primary and secondary causes of death

The primary causes of death (*n* = 298) were medical emergency diseases: these were cardiovascular diseases 83 (27.8%), respiratory diseases 78 (26.2%), infectious diseases 76 (25.5%) and hematological diseases 32 (10.7%).Surgical and accidental cases were contributed 7.4% and 4.4% for pediatric morality respectively (Table 2).

**Table 2:** Frequency distribution of primary and secondary causes of death in PED at TASTH, Addis Ababa, from 2012 to 2016 inclusive

Variables	Frequency	Percentage (%)
Medical emergency diseases (*N* = 298 (88.25%))		
Respiratory diseases (*n* = 78)		
Severe pneumonia	60	76.9
Tuberculosis	13	16.7
Others	5	6.4
Infectious diseases (*n* = 76)		
Sepsis	40	52.6
Meningitis	34	44.7
Malaria	2	2.7
Cardiovascular diseases (*n* = 83)		
Congestive heart failure	46	55.5
Hypovolemic shock	20	24.1
Septic shock	6	7.2
Pulmonary hypertension	6	7.2
Cardiogenic shock	3	3.6
Anaphylactic shock	2	2.4
Hematological diseases (*n* = 32)		
Hematological malignancy	15	46.9
Severe anemia	14	43.8
Hemophilia	3	9.3
Digestive diseases (*n* = 12)		
Diarrheal diseases	7	58.3
Hepatic encephalopathy	5	41.7
Renal diseases (*n* = 9)		
Renal failure (acute and chronic)	8	88.9
Nephrotic syndrome	1	11.1
Neurological diseases (*n* = 8)		
Seizure disorder	5	62.5
Guillain-Barré syndrome	2	25.0
Intra cranial pressure	1	12.5
Surgical cases (*n* = 25)		
Abdominal mass	9	36.0
Small bowel obstruction	6	24.0
Large bowel obstruction	4	16.0
Intussusception	3	12.0
Others	3	12.0
Accidental/unintentional injuries (*N* = 15)		
Severe traumatic brain injury	5	33.3
Other than head injury	8	53.4
Burn	2	13.3
Secondary causes of death (*n* = 146)		
Malnutrition	60	41.1
Congenital heart defect	37	25.4
Down syndrome	21	14.4
Malignant tumors	10	6.8
Low birth weight	7	4.8
Others	11	7.5

Note: Others: -For surgical cases (hydrocephalus, abdominal herniae), for respiratory diseases (ARDS, asthma), for secondary cause (prematurity, HIV, diabetes mellitus)

Two third of abdominal masses were malignancy-related masses; half of these were Wilm’s tumors. Other presentations included renal failure (acute and/or chronic); severe traumatic brain injury (TBI) (epidural and/or subdural hematoma), increased intra-cranial pressure (ICP), and abdominal herniae after previous abdominal surgery.

Overall almost half of the primary causes of death had co-morbidities with secondary causes of mortality (including malnutrition, congenital heart defect, Down syndrome, malignant tumors, and low birth weight). Malnutrition and congestive heart disease were the most common co-morbidities associated with the primary causes of death. Prematurity (3.4%), HIV/AIDS (2.7%) and diabetes mellitus (1.4%) were other less common co-morbidities (Table 2).

The top ten cause of mortality were pneumonia 17.8%, congestive heart failure 13.6%, sepsis 11.8%, meningitis 10.1%, hypovolemic shock 6%, hematological malignancy 4.4%, anemia 4.1%, tuberculosis 3.9%, abdominal mass 2.7% and renal failure 2.4% (Table 3).

**Table 3:** Distribution of age category groups with top ten causes of mortality in PED at TASTH, Addis Ababa, from 2012 to 2016 inclusive

Top ten causes of death	Age category			
	Neonate n (%)	Infant n (%)	Preschool age n (%)	School age n (%)
Pneumonia (*n* = 60)	6 (10.5)	26 (35.6)	20 (26.7)	8 (14.8)
Congestive heart failure (*n* = 46)	4 (7.0)	17 (23.3)	12 (16.0)	13 (24.1)
Sepsis (*n* = 40)	29 (50.9)	3 (4.1)	7 (9.3)	1 (1.9)
Meningitis (*n* = 34)	14 (24.6)	13 (17.8)	5 (6.7)	2 (3.7)
Hypovolemic shock (*n* = 20)	3 (5.3)	5 (6.8)	6 (8.0)	6 (11.1)
Hematological malignancy (n = 15)	–	–	8 (10.7)	7 (13.0)
Anemia (*n* = 14)	1 (1.8)	5 (6.8)	1 (1.3)	7 (13.0)
Tuberculosis (*n* = 13)	–	–	6 (8.0)	7 (13.0)
Abdominal mass (n = 9)	–	1 (1.4)	7 (9.3)	1 (1.9)
Renal failure (*n* = 8)	–	3 (4.1)	3 (4.0)	2 (3.7)

The top causes for neonatal deaths were late-onset sepsis (50.9%) and meningitis (24.6%), while in infants, pneumonia (35.6%) and congestive heart failure (23.3%) were the main causes of death. This was similar in the preschool age children with pneumonia at 26.7% and congestive heart failure at 16%. On the other hand, congestive heart failure was the most common cause of death for the school age group, followed by pneumonia and hematological malignancy. There were no neonatal cases of tuberculosis, renal failure, abdominal mass and hematological malignancy (Table 3).

Notably, malnutrition was a significant co-morbidity with all top ten causes of death in pediatric emergency. A congenital heart defect commonly contributed to death from congestive heart failure and pneumonia. HIV/AIDS was co-morbidity with tuberculosis and pneumonia while low birth weights were related with the late onset of sepsis and meningitis (Table 4).

**Table 4:** Top ten and co-morbidity cases of death in PED at TASTH, Addis Ababa, from 2012 to 2016 inclusive

Top ten diseases	Secondary causes of mortality (N (%))							
	Malnutrition	Congenital heart defect	Down syndrome	Malignancy tumor	Low birth weight	Prematurity	HIV	Diabetes mellitus
Pneumonia (*n* = 30)	17 (56.7)	7 (23.3)	2 (6.7)	1 (3.3)		1 (3.3)	2 (6.7)	
Congestive heart failure (*n* = 43)	9 (20.9)	21 (48.8)	10 (23.3)	2 (4.6)			1 (2.4)	
Sepsis (*n* = 11)	4 (36.4)	4 (36.3)	1 (9.1)			1 (9.1)		1 (9.1)
Meningitis (*n* = 8)	1 (12.5)		3 (37.5)		2 (25)	2 (25)		
Hypovolemic shock (*n* = 8)	3 (37.5)	2 (25)	3 (37.5)					
Hematological malignancy (*n* = 11)	7 (63.6)			4 (36.4)				
Anemia (*n* = 4)	3 (75.0)				1 (25.0)			
Tuberculosis (*n* = 5)	4 (80.0)						1 (20.0)	
Abdominal mass (*n* = 1)				1 (100)				
Renal failure (*n* = 1)	1 (100)							

Table 5 documents the result of crude and adjusted odds ratios after logistic regression. In univariate logistic regression analysis only six variables fulfilled the criteria of *p*-values less than 0.2. These were age, duration of signs and symptoms, sign and symptoms, hematological malignancy, diarrheal disease and malnutrition.

**Table 5:** Factors (crude and adjusted odds ratios and confidence intervals) associated with early pediatric mortality in PED at TASTH, Addis Ababa from 2012 to 2016 inclusive

Variables	Mortality		COR (CI, 95%)	AOR (CI, 95%)	*p* value
	≤24 h	> 24 h			
Age					
Neonate	24	45	1.47 (0.73–2.98)	0.96 (0.44–2.09)	0.982
Infant	31	61	1.40 (0.73–2.72)	1.15 (0.56–2.36)	0.912
Preschool age	34	64	1.47 (0.77–2.81)	1.57 (0.77–3.21)	0.187
School age	21	58	1.00	1.00	
Duration of signs and symptoms					
≤ two days	57	77	1.00	1.00	
> two days	53	151	2.11 (1.33–3.35)	3.22 (1.34–7.73)**	0.004
Sign and symptoms					
Fast breathing	18	28	2.21 (1.00–4.88)	2.78 (1.19–6.49)*	0.020
Fever	15	20	2.58 (1.10–6.05	3.17 (1.28–7.86)*	0.019
Vomiting	9	23	1.35 (0.53–3.42)	1.48 (0.55–4.03)	0.573
Cough	5	29	0.59 (0.20–1.76)	0.70 (0.23–2.18)	0.625
Shortness of breath	45	66	2.35 (1.23–4.49)	2.45 (1.22–4.91)**	0.006
Other diseases	18	62	1.00	1.00	
Hematological malignancy					
Yes	4	11	0.74 (0.23–2.39)	1.08 (0.29–4.03)	0.814
No	106	217	1.00	1.00	
Diarrheal disease					
Yes	85	208	3.06 (1.61–5.80)	3.36 (1.69–6.67)**	0.009
No	25	20	1.00	1.00	
Malnutrition					
Yes	24	67	1.49 (0.87–2.55)	1.43 (0.79–2.57)	0.226
No	86	161	1.00	1.00	

Other signs and symptoms included respiratory distress, swelling, coma, convulsion, grunting, abdominal pain, distension, headache, failure to suck

Note: -* Significant association (*p*-value < 0.05), −** significant association (p-value < 0.01), Hosmer and Lemeshow goodness of fit test = 0.985

*COR* crude odds ratio, *AOR* adjusted odds ratio

In the multivariate logistic regression, shortness of breath, fast breathing, fever, late onset of signs and symptoms (> two days), and diarrheal disease were significantly associated with early rather than late pediatric mortality.

Children with shortness of breath were more likely to have an early death after emergency department admission than those developing other signs and symptoms [AOR = 2.45, 95% CI (1.22–4.91)]. Those participants presenting to an emergency unit after two days of illness had three times greater odds of early mortality compared to those who presented earlier[AOR = 3.22, 95% CI (1.34–7.73)]. Diarrheal disease and fever were also a risk factor for early child mortality (Table 5).

## Discussion

Children presenting to our emergency department, had a mortality rate of 4.1%. The incidence of pediatric mortality in this study was lower than the previous studies conducted in Ethiopia [23–26]. This decrease may be due to improvements in the maternal and child urban health care settings. Despite this low mortality rate, early death less than 24 h after admission 107 (31.7%) was still high albeit, lower than other studies [18, 19, 26, 27].

The majority of pediatric emergency mortality in hospitals was due to preventable diseases within 24 h of admission [12]. This may be due to poor health care seeking behavior, delay in referral, using traditional medicine, and poverty [28]. A study done in Sub-Saharan countries on care-seeking behaviors related to respiratory illness were found that only 30% of Ethiopian children with suspected pneumonia were taken to a health care settings; this was the lowest proportion in the six analyzed countries [29]. Another study identified multiple factors influencing care-seeking behaviors in Ethiopian children including lack of knowledge, delay in recognition of illness severity and household income [30]. The main causes of neonatal death were late-onset sepsis (50.9%), meningitis (24.6%), and pneumonia (10.5%). Previous studies done in Nigeria and Benin identified high rates of sepsis in this age group [20, 21, 31], which may be due to unclean cord care practices, traditional birth attendant, polluted atmosphere and poor health education among parents [32].

We found that the primary causes of death for the infant and pre-school age groups at TASTH were pneumonia, congestive heart failure, meningitis, and sepsis and hypovolemic shock. This is similar to previous work in China, Nigeria, Ghana, India and a World Health Organization (WHO) report [19, 20, 22, 33, 34]. In Ethiopia, pneumonia is an important public health problem for all children, and creates a significant burden of childhood mortality [18, 35]. A health extension program package focused on disease prevention and health education targeting antibiotic treatment for childhood pneumonia might be a solution to decrease deaths from pneumonia. However, there are some challenges in promoting a health extension program package due to knowledge gaps of health extension workers such as misdiagnosis, negligence and inappropriate referrals [36, 37]. These problems need to be addressed and improved because pneumonia contributes to the high early pediatric mortality in developing countries [38].

Our study found that congestive heart failure and hematological malignancy were top primary causes of school age group mortality. This is consistent with findings in China and Nigeria [19–21], but quite different to other studies in Nigeria, Ethiopia and sub-Saharan Africa countries which suggested a smaller role for these conditions [26, 39, 40]. This disparity may be due to lack of cardiac and pediatric oncology services in developing countries. Many low- and middle-income countries lack pediatric cardiac care programs, resulting insignificant mortality from congenital heart diseases [41]. Other possible causes include lack of primary care, screening and health follow-up in low income countries. However, even with early diagnosis, accesses to expensive chemotherapy agents and/or specialized cardiac surgery are also severely limited.

Our study found that the most five common presenting symptoms of children who died within the PED were shortness of breath, fast breathing, fever, vomiting, and cough. Shortness of breath, fever and fast breathing were associated with early mortality when compared to the other common presenting symptom. These findings are similar to the studies found in Ghana and South East Nigeria [22, 42].

These common signs and symptoms are usually identified by emergent assessment of airway, breathing and circulation [13, 14, 43]. Consequently, immediate treatment and management is critical, particularly for airway obstruction that leads to severe illness and death when left untreated. Early triage assessment and identification of signs of critical illness, and rapid initiation of appropriate treatment should be priorities for all hospitals providing emergency care for children.

Malnutrition and diarrhea were common co-morbid conditions associated with the primary causes of death. This is consistent with studies conducted in Kenya, Ghana [22, 44] and Ethiopia [18, 26]. Additionally, in our study diarrheal disease was significantly associated with pediatric mortality. In Africa, many studies have identified diarrheal disease to be a significant cause of death in childhood [20, 22, 25, 44–51].

Malnutrition was a co-morbid condition in one-third of pediatric deaths. Micronutrient initiative programs and multi-sector collaboration may be useful interventions to improve community awareness of the importance of balanced nutrition. However, difficulty accessing or affording food is a significant challenge for large numbers of African children [52].

Our study has potential limitations. This includes the retrospective study design, and the reliance on interpretation of documentation within the medical record. In some cases, it was difficult to obtain adequate study information. Unfortunately, we were unable to collect data on almost one-third of all cases due to incomplete documentation (95, 19.1%) of the patient’s medical history, loss of the medical chart (66, 13.2%) and one patient who had multiple diagnoses recorded, rendering it difficult to identify primary and secondary causes of mortality.

## Conclusion

The total mortality rate of children in this study was 4.1% with a high proportion (31.7%) of early mortality. Pneumonia, congestive heart failure, sepsis, meningitis, late-onset sepsis and hematological diseases were the most common causes of death in children presenting to our emergency department. A delay in presentation of more than 48 h, diarrheal diseases and shortness of breath were significantly associated with early pediatric mortality. Almost all mortality was due to preventable diseases, which can be controlled with minimum resources and quality care provision. We were unable to extract data for a significant proportion of patients due to limitations of and/or missing medical documentation. Efficient, evidence-based triage and intervention by trained ED staff may improve child mortality. Further longitudinal studies on pediatric emergency patients in the African setting are warranted.

## Abbreviations

AIDS: Acquired immune deficiency syndrome
ARDS: Acute respiratory disease syndrome
CHF: Congestive heart failure
ED: emergency department
GBS: Guillain-Barré syndrome
HIV: Human immunodeficiency virus
HTN: Hypertension
LBW: Low birth weight
PED: Pediatric emergency department
SOB: Shortness of breath
TASTH: Tikur Anbessa Specialized Tertiary Hospital
WHO: World Health Organization
